# NMR spectroscopy for metabolomics in the living system: recent progress and future challenges

**DOI:** 10.1007/s00216-024-05137-8

**Published:** 2024-01-19

**Authors:** Yun Peng, Zeting Zhang, Lichun He, Conggang Li, Maili Liu

**Affiliations:** 1 State Key Laboratory of Magnetic Resonance and Atomic Molecular Physics, National Center for Magnetic Resonance in Wuhan, Innovation Academy for Precision Measurement Science and Technology, Chinese Academy of Sciences, Wuhan, 430071 China; 2 Optics Valley Laboratory, Wuhan, 430074 Hubei China

**Keywords:** Bioanalytical methods, NMR/ESR, Metabolomics

## Abstract

Metabolism is a fundamental process that underlies human health and diseases. Nuclear magnetic resonance (NMR) techniques offer a powerful approach to identify metabolic processes and track the flux of metabolites at the molecular level in living systems. An in vitro study through in-cell NMR tracks metabolites in real time and investigates protein structures and dynamics in a state close to their most natural environment. This technique characterizes metabolites and proteins involved in metabolic pathways in prokaryotic and eukaryotic cells. In vivo magnetic resonance spectroscopy (MRS) enables whole-organism metabolic monitoring by visualizing the spatial distribution of metabolites and targeted proteins. One limitation of these NMR techniques is the sensitivity, for which a possible improved approach is through isotopic enrichment or hyperpolarization methods, including dynamic nuclear polarization (DNP) and parahydrogen-induced polarization (PHIP). DNP involves the transfer of high polarization from electronic spins of radicals to surrounding nuclear spins for signal enhancements, allowing the detection of low-abundance metabolites and real-time monitoring of metabolic activities. PHIP enables the transfer of nuclear spin polarization from parahydrogen to other nuclei for signal enhancements, particularly in proton NMR, and has been applied in studies of enzymatic reactions and cell signaling. This review provides an overview of in-cell NMR, in vivo MRS, and hyperpolarization techniques, highlighting their applications in metabolic studies and discussing challenges and future perspectives.

**Graphical abstract:**

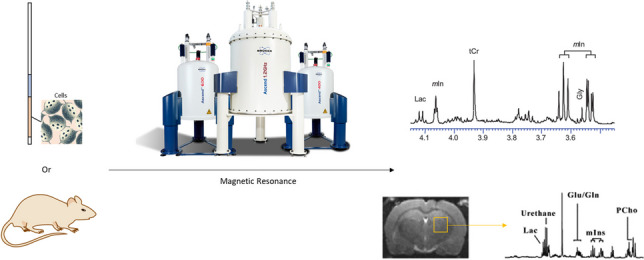

## Introduction

Metabolism occurs within the cells of a living organism and provides energy for vital processes. The study of metabolism reveals insights into molecular and energy processes of living organisms and essentially underlies human health and diseases [1]. Nuclear magnetic resonance (NMR) techniques offer a powerful approach for metabolism studies at the molecular level, leading to the identification of metabolic processes and tracking the flux of metabolites in living systems, both in vitro and in vivo.

In this review, we first summarize the in vitro study of metabolism through in-cell NMR. This technique tracks metabolites in real time and in a state close to their natural environment. We then discuss in vivo magnetic resonance spectroscopy (MRS), which enables whole-organism metabolic monitoring by visualizing the spatial distribution of metabolites and targeted proteins. One limitation of these NMR techniques is the sensitivity since metabolites in natural environments are usually kept in low concentration. In this review, we also highlight a couple of hyperpolarization methods, including dynamic nuclear polarization (DNP) and parahydrogen-induced polarization (PHIP), that assist in metabolic studies. Hyperpolarization involves hyperpolarizing electrons or other nuclei, and transfer of this spin polarization to nuclei of interest [2]. DNP deploys polarization transfer from electron spins to nuclear spins of interest, and PHIP utilizes parahydrogen for polarization transfer to enhance the detection signal. Both of the hyperpolarization methods have been applied in studies of enzymatic reactions, cell signaling, disease diagnosis, and related fields. Overall, we demonstrate the essential role of in-cell NMR and in vivo MRS in different areas of metabolism studies.

## Metabolism study via in-cell NMR

Nuclear magnetic resonance (NMR) can provide information about molecules under physiological conditions because it is non-invasive. In the past decades, in-cell NMR has emerged as a powerful technique for the observation of metabolites and macromolecules directly inside living cells with atomic resolution, thus providing insights into metabolic pathways and molecular mechanisms in a physiologically relevant environment. In cells, the concentration of macromolecules is as high as 300 g/L [3, 4]. To distinguish from other cellular components, the molecule can be labeled with NMR active nuclei such as ^13^C, ^31^P, ^15^N, and ^19^F. Due to the sensitivity of nuclear spin properties to the local chemical environment, as well as the quantitative and non-invasive nature of NMR, in-cell NMR spectroscopy can provide structural and quantitative information about the behavior of molecules, as well as information on the temporal resolution inside living cells.

In the field of conventional metabolomics, the metabolite profile is measured for a steady state at a given time point. In-cell NMR makes it possible to investigate metabolism in living cells, thereby profiling the metabolic dynamic patterns by recording a time series of successive spectra on a single sample without the need for cell extraction, as well as determining metabolism close to living conditions. However, studies of metabolism in living cells are more technically challenging than in vitro studies. In addition to the inherently poor sensitivity of NMR, decreased cell integrity caused by long experiment time and severe signal broadening are also major factors limiting standard NMR measurements of metabolism in living cells. Over the past few decades, numerous efforts focused on boosting the sensitivity and resolution and speed of data acquisition have enabled the identification and quantitation of a large number of metabolites in living cells, and the monitoring of metabolism in real time. We review here a series of NMR-based methodological advancements in recent years and successful applications for probing metabolism in living cells.

### High-resolution magic angle spinning (HR-MAS) NMR

**Fig. 1:**
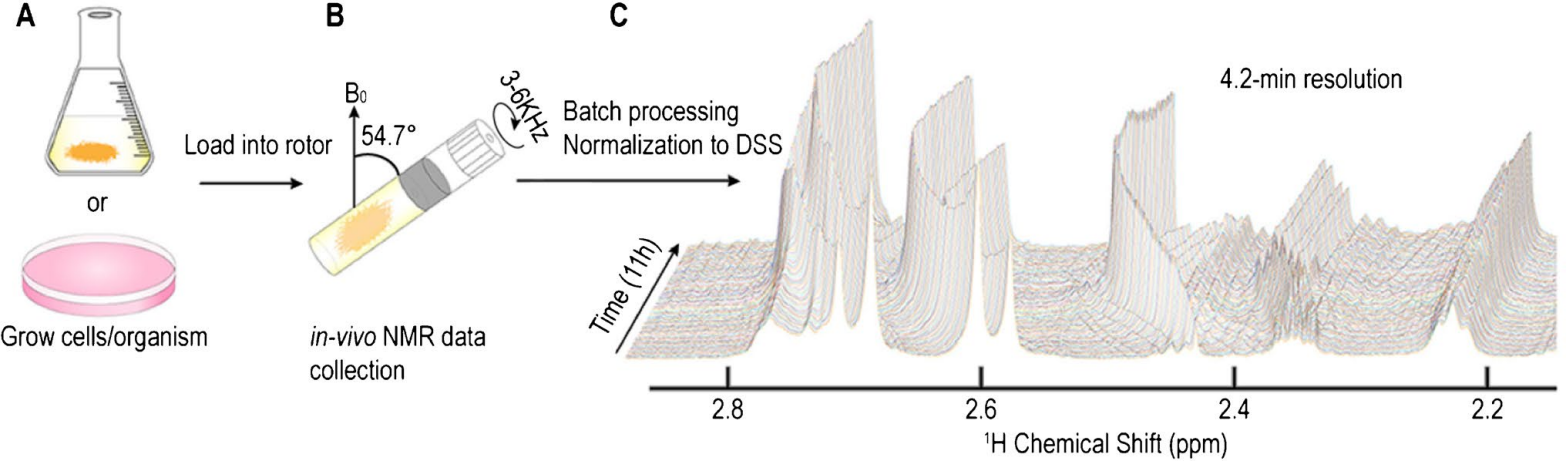
Sample preparation and analysis for continuous in vivo monitoring of metabolism by HR-MAS NMR experiments. Cells or organisms were grown in nutrient-rich media (**A**) and transferred to the HR-MAS rotor (**B**). NMR data of *Neurospora crassa* were collected over 11 h with temporal resolution at 4.23 min (**C**) [5]. Adapted with permission from ref 5, Copyright 2019 Frontiers Media S.A

For samples of highly complex bio-mixtures, the heterogeneity in the magnetic susceptibility across the sample and restricted motion of mobile species result in severe line broadening of the NMR resonances, which greatly affects the identification and quantification of metabolites using traditional NMR techniques. ^1^H detected high-resolution magic angle spinning (HR-MAS) NMR, which has now emerged as a powerful tool for the investigation of heterogeneous samples such as living cells, tissues, and living organisms [5–11]. Spinning of the sample at an angle of 54.74° (the magic angle) with respect to the static magnetic field B_0_ reduced the line-broadening effects and hence yielded well-resolved NMR signals [12]. The rotor spinning rate of HR-MAS is relatively low (ca. 3–6 kHz) compared to that of solid-state MAS NMR, thus maintaining the integrity of cells while removing the spinning side bands from interested regions of the spectra (Fig. 1).

The potential for using HR-MAS NMR as a non-invasive tool for metabolism research in living cells has been demonstrated by several studies. Using HR-MAS ^1^H NMR, the metabolic composition was investigated directly on whole cells of marine unicellular microalgae [13]. Using 1D and 2D HR MAS NMR, Righi et al. [14] quantified several informative metabolic molecules that are important in peptidoglycan synthesis, providing a complete metabolic profile of *Pseudomonas aeruginosa*. Successful application of HR-MAS NMR in the study of microalgae cell and bacteria metabolism have shown that it improves spectral resolution and provides a powerful tool for metabolism studies in intact cells. However, the potential for cell lysis results from rapid spinning can be a serious problem for fragile cells. Meanwhile, the inherent low sensitivity and thus need for large amounts of samples is one of the major drawbacks of NMR. These are not conducive to the studies on living cells. One approach to solve these problems is the use of the emerging micro-NMR technology, high-resolution magic-angle coil spinning (HR-MACS), to generate high-resolution spectra from a small amount of samples at a low spinning rate [15]. A secondary tuned circuit and a simple and robust rotor insert were designed to fit inside a standard MAS sample rotor, thus converting the MAS probe into a micro-MAS probe for analysis of small-volume samples [16, 17]. Alan et al. applied HR-MACS for a high-sensitivity and high-resolution ^1^H micro-NMR-based metabolomics study on yeast cells. The combination of slow sample spinning (300 Hz) and small sample diameter minimizes the centrifugal forces exerted upon the cells under rotation, thus enabling the analysis of more fragile cells. The study comprehensively profiles the metabolite constituents (with about 20 metabolites) of samples containing only 19 million intact yeast cells (~ 250 nl) and distinguishes their metabolome at various stages of growth or under osmotic stress [15]. Thus, HR-MACS offers a powerful approach to high-quality micro-NMR analysis on metabolism in living cells.

**Fig. 2:**
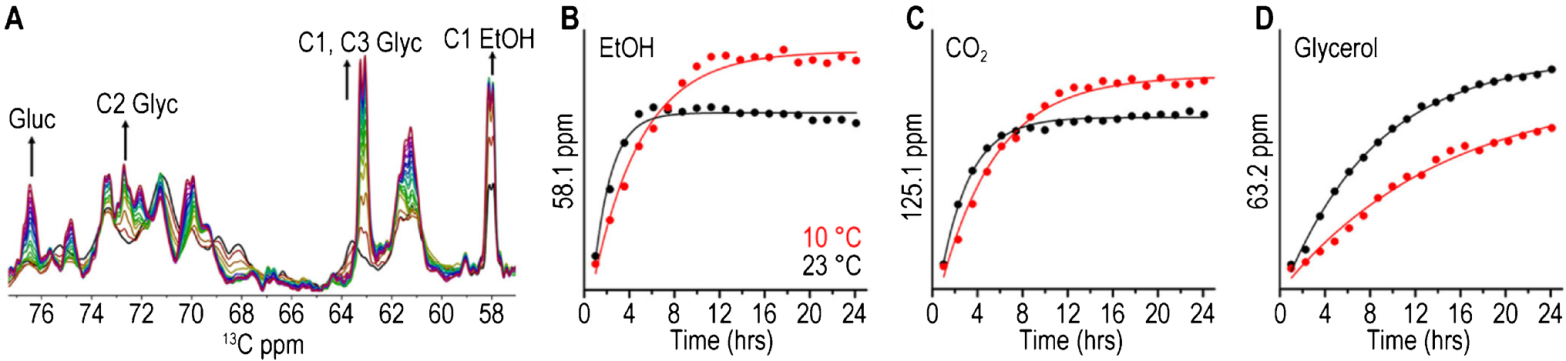
Time-dependent in-cell changes monitored by NMR. **A** Time-dependent series of DP ^13^C ssNMR spectra of *Cr.* cells at 23 °C followed by a continuous 24-h measurement. **B**–**D** DP NMR signal intensities at 10 °C (red) and 23 °C (black) as a function of time for ethanol (58.1 ppm, **B**), CO2 (121.1 ppm, **C**), and glycerol (63.2 ppm, **D**) [18]. Adapted with permission from ref 18, Copyright 2022 John Wiley and Sons

In addition to using HR-MAS to study small metabolic molecules in living cells, a recent work reported a real-time multi-phase MAS NMR approach with dynamics spectral editing to simultaneously monitor microbial processes of fermentation, lipid metabolism, and structural dynamic changes in live microalgae cells [18]. The unicellular green algae *Chlamydomonas reinhardtii* (Cr.) cells were uniformly ^13^C labeled and subjected to dark, anoxia conditions that trigger fermentation, by concentrating the cell suspensions in MAS NMR rotors, and subsequently followed time-dependent in-cell changes by solid-state ^13^C NMR spectroscopy. By using ^13^C MAS NMR-based direct polarization (DP) single-pulse experiments, the processes of fermentation and lipid metabolism were observed in a time-dependent manner (Fig. 2). Dynamic-based spectral editing cross-polarization (CP) and insensitive nucleus-enhanced polarization-transfer (INEPT) experiments were employed to provide further insight into structural dynamics changes of lipid membranes and carbohydrate macrostructures with micro- to millisecond dynamics and (sub)nanosecond dynamics, respectively. They found that in parallel with the fermentation processes and accumulation of FFAs, rapid loss of rigid lipid structures was observed, suggesting that incubating Microbial Cr. Cell suspensions under dark and anoxia conditions results in the switch to fermentation metabolism and finally breakdown of lipid membranes [18].

### Signal enhancement using dissolution dynamic nuclear polarization (DNP) for in-cell NMR

NMR metabolomic studies based on ^1^H 1D spectroscopy are severely limited by signal overlap. Although ^13^C NMR benefits from a large signal dispersion combined with relatively narrow spectral lines, it is barely used to detect transient pathway intermediates and rapid transformations inside living cells, mainly due to its poor sensitivity and temporal resolution. The recent development of dissolution dynamic nuclear polarization (DNP) can enhance single-scan liquid-state ^13^C NMR signals by more than 10,000-fold [19]. Nuclear polarization is generated by transferring unpaired electron polarization to the observed nuclei by microwave irradiation at ~ 1–1.5 K [20]. The hyperpolarized sample is then rapidly melted and transferred to a detection magnet for the NMR measurement, yielding a sufficiently enhanced signal to follow metabolic processes in living systems with high temporal resolution.

In combination with a series of hyperpolarized metabolites enriched in ^13^C at slow-relaxing nuclear sites, the utility of hyperpolarization-based NMR analysis has been demonstrated to study non-invasively molecular metabolism in living systems in numerous recent studies. In 2011, Meier et al. [21] investigated the metabolic pathway in living yeast cells by DNP-NMR; [U-^2^H, U-^13^C] glucose and [2-^13^C] fructose were used to provide dietary substrates containing non-protonated ^13^C nuclei with sufficiently long T_1_ times for prolonging the hyperpolarized state. A series of ^13^C NMR spectra were recorded after the injection of DNP-enhanced dietary monosaccharide into the cell suspension. The real-time DNP-NMR assay follows an entire pathway with more than twelve enzyme-catalyzed transformations from glucose to the end products ethanol and CO_2_ via various transient pathway intermediates. Various glycolytic intermediates such as fructose-1,6-bisphosphate (Frc-1,6BP), dihydroxyacetone phosphate (DHAP), and pyruvate can be detected and tracked for dDNP signal enhancement. Accumulation of 6-phosphogluconate (6-PG) was also observed as the main intermediate of the oxidative pentose phosphate pathway. They show that the use of DNP-enhanced nutrients in conjunction with NMR spectroscopic detection permits the visualization of entire pathways in living cells [21].

In 2013, Harris et al. [22] applied dissolution DNP to study the glycolytic metabolism in living perfused human breast cancer cells. The perdeuterated [U-^13^C, ^2^H_7_]-glucose was used to increase the T1 value of its carbon atoms for prolonging the hyperpolarized state for NMR measurement. Hyperpolarized [U-^13^C, ^2^H_7_]-glucose was injected into the cell perfusion system, followed by NMR measurement of hyperpolarized ^13^C glucose metabolism in living cells. The high temporal resolution of hyperpolarized NMR metabolic measurements enabled the non-invasive and direct observation of a number of glycolytic intermediates, as well as the determination of the initial glycolytic rate in living cancer cells. More importantly, the reaction intermediates whose concentrations are too low for direct detection even with the enhanced DNP signal were observed indirectly by applying saturation transfer (ST) strategies. Therefore, in addition to increased sensitivity by dissolution DNP, the ST experiments provide additional signal sensitivity for the identification of low-concentration metabolic intermediates [22].

In 2019, Jensen et al. [23] reported an approach combining hyperpolarized in-cell NMR with simulation to probe redox-dependent metabolic control which occurs on a time scale of seconds. Isotope-enriched metabolic substrates [U-^13^C, U-^2^H]-glucose, [2-^13^C]-fructose, and [6-^13^C,6,6’-^2^H_2_]-glucose were hyperpolarized using dissolution DNP and then employed to track the conversion of carbohydrate spins to glycolytic intermediates and end products by recording a series of ^13^C NMR spectra in living *S. cerevisiae*. They found that in pre-steady-state glycolysis, bottlenecks shift from downstream to upstream in the pathway within a few seconds. The application of hyperpolarized in-cell NMR provides a promising method for characterizing metabolic pathways and gaining more knowledge about mechanistic details [23].

### 2D in-cell NMR spectroscopy

The use of stable isotopically enriched tracers is indispensable for elucidating metabolic pathways and pathway dynamics. The simplicity of 1D NMR in terms of acquisition and processing is an advantage. However, the narrow frequency range of 1H results in significant resonance overlap, while the low sensitivity of ^13^C nuclei limits the wide application of ^13^C-detected NMR in metabolism studies [24]. In recent years, the ^1^H-detected 2D NMR technique, heteronuclear single-quantum correlation (HSQC), that offers significantly enhanced sensitivity and an improved resolution has been used in metabolic studies in living cells. In 2015, Wen et al. [25] used ^13^C_6_-glucose and heteronuclear 2D NMR for sensitive and high-resolution real-time monitoring of metabolites from living cancer cells, enabling metabolomics differentiation between cancer and normal cells on the basis of time-dependent changes in metabolite concentration, as well as evaluation of the metabolic effects of anticancer agent. In addition, the oxidation of glutathione by a reactive oxygen species (ROS)–producing agent was monitored in real time using [U-^13^C]-labeled cysteine with heteronuclear NMR, and a possible new mechanism of temozolomide (TMZ) resistance involving ROS neutralization was identified in brain cancer cells [26].

In stable isotope tracer studies, isotope labels are incorporated into specific atomic positions of given metabolites depending on the transformation pathways. The label positions combined with the number of isotope labels are crucial for the reconstruction of metabolic pathways and networks [27]. NMR spectroscopy is uniquely able to differentiate various isotopomers and track stable isotope labeling at specific atomic positions, while MS-based approaches cannot easily distinguish between positional isotopomers. Although isotope-edited two-dimensional NMR has advantages in those studies, the poor resolution in the indirectly detected 13C dimension hinders its widespread application in the isotopomer analysis [28]. In addition, multidimensional experiments are very time-expensive due to the costly sampling of evolution time space, which is detrimental to studies on living cells. In 2017, Lee et al. [29] reported a NUS-based 2D heteronuclear single-quantum coherence (NUS HSQC) approach to measure metabolic isotopomers in living cells. NUS has been developed to collect only a fraction of the data points and convert the NUS data afterwards into a full signal using mathematical reconstruction algorithms. NUS enables faster collection of NMR spectra and enhances the resolution of the indirect dimension, thus is particularly useful for isotopomer studies in living cells [30]. With hmsIST [31], one of the fastest NUS approaches, Lee et al. [29] demonstrate that 2D NUS HSQC with 25% sampling density can give high-resolution 2D data with very good resolution of carbon isotopomer multiplets in L1201 mouse leukemia cells labeled with ^13^C acetate. The approach allows detailed metabolomics flux analysis including metabolic pathway usages and TCA cycle efficiency, and reveals the metabolic information about position-dependent isotopomer patterns in fatty acids [29]. The high sensitivity and resolution along with the application to live cells should make the NUS HSQC approach attractive in studying carbon flux information in metabolic studies.

### Metabolism studies in primary patient cells

Metabolic-targeted therapy is becoming a core research area in the development of various cancer therapeutic approaches. Understanding the metabolism of cancer cells is critical to exploring new therapeutic targets [27, 32]. Using 1D ^1^H-NMR spectra, Koczula et al. [33] studied metabolism in living primary patient cancer cells in real time. Blood-derived chronic lymphoid leukemia (CLL) cells were embedded in a dilute agarose matrix to prevent sedimentation thus preserving the homogeneity of the sample. By recording a series of ^1^H NMR spectra, metabolic response to hypoxia was measured with a time resolution of 5–8 min. It is found that CLL cells display remarkable plasticity of metabolic adaptation and functional changes in the protective utilization of pyruvate [33]. More recently, Islam et al. reported an automated real-time NMR approach that enables the study of cellular metabolism on sensitive primary patient cells in a real-time manner with lower cell numbers. The acute myeloid leukemia (AML) cells, which are highly sensitive to the culture condition, were embedded in methylcellulose to achieve improved cell viability compared to that in agarose. This approach needs only a small number of cells (approximately 5 × 10^5^ cells or even fewer) and enables monitoring the cellular metabolism by observing the consumption/production of different metabolites over several kinetic data points of up to 48 h [34]. These studies show that NMR is capable of monitoring cellular metabolism on primary patient cells at temporal resolution, which has great potential for applications in personalized medicine.

### In-organelle NMR metabolism

NMR spectroscopy is a powerful method that can measure dynamic changes in metabolism in living cells. However, subcellular spatially resolved information is lacking at the whole-cell level in in-cell NMR studies. Investigating metabolism in specific organelle alleviates interferences from other cellular components and hence promises unique insights into the metabolism of specific organelles. Mitochondria are important subcellular organelles that carry out fundamental functions in eukaryotic cells. They are best known as the “powerhouse” of the cell as they produce respiratory ATP. With the recognition of altered mitochondrial metabolism as the hallmark of cancers, the investigation of mitochondrial metabolism is gaining increased interest [30, 35]. Since the development of methods for isolating functional mitochondria, a series of studies on metabolism have been performed in isolated mitochondria. Using 1D ^13^C NMR, Offermann et al. [36] firstly monitored the Krebs cycle in isolated heart mitochondria in 1992, while Shieh et al. [37] investigated the ibuprofen metabolism in rat liver mitochondria in real time. The real-time metabolism of malate was also monitored in mitochondria isolated from bovine adrenocortical using ^13^C NMR spectroscopy [38]. Although real-time in-organelle metabolic measurements are successfully achieved by 1D ^13^C NMR spectroscopy, the method suffers from extremely low sensitivity with signal overlap. In recent years, the 2D NMR technique has been used for metabolic studies in mitochondria. Employing heteronuclear 2D NMR spectroscopy and ^13^C_3_-pyruvate, Xu et al. [39] investigate the pyruvate metabolism in live human mitochondria in real time. The approach identified acetyl phosphate as a metabolic intermediate and allowed for the estimation of pyruvate dehydrogenase (PDH) enzyme activity [39]. More recently, by recording the sensitivity-enhanced ^1^H-^13^C HSQC spectra using live mitochondria isolated from muscle cells derived from mouse skeletal myoblasts (C2C12), Gowda et al. [40] found that lactate is produced from pyruvate inside the mitochondria, which is known to occur only in the cytoplasm. The in-organelle NMR approach should be very useful in studies on organelle-specific contributions to metabolism in health and diseases and can also be extended to other organelle-specific metabolic studies.

## In vivo magnetic resonance spectroscopy (MRS)

In vivo MRS follows the principle of the NMR technique and uses magnetic resonance chemical displacement to determine the molecular composition and concentration in living tissues. Due to the non-invasive advantage of NMR, MRS is currently the only non-invasive method that can detect metabolites and contribute to metabolism studies in vivo. MRS experiments involve transmitting a pulse to excite the nuclei in a given volume and then receiving returning signals. The resulting spectrum is a plot of signal intensity versus frequency over the excited range. Compounds of interest exhibit unique special signatures including invariant quantities, chemical shift positions, and relative peak intensities. Peaks observed in the spectrum are attributed to specific metabolites according to the chemical shift, and a quantification procedure based on the integration of assigned peaks and the calibration of concentration ascertains quantitative information associated with corresponding metabolites [41]. Analysis of the spectrum can therefore address chemicals in their presence and their respective concentrations when peak areas are correctly calibrated with respect to standard references [42]. These features make MRS a useful analytical tool for chemistry and clinical diagnosis including oncology and radiology. The frequencies of MRS equipment can be varied to measure resonances from ^1^H, ^13^C, ^19^F, ^31^P, and even ^23^Na to detect corresponding metabolites. Among them, ^1^H is by far the most widely monitored nucleus, because it is very abundant and provides a strong magnetic resonance signal [43]. Compared to NMR, MRS can track metabolites of interest in selected regions within the living tissue and human body, and detect metabolic changes linked to the development and progression of diseases focused [44–47]. MRS is usually applied in parallel with the magnetic resonance imaging (MRI) technique. Aside from images of the internal structure of the organism provided by MRI, chemical compositions of tissues, especially representative metabolites and other compounds signaling diseases like cancer, can be identified and quantified via MRS.

### Development and uniqueness of MRS

A decade after the independent observation of NMR in bulk materials by Bloch et al. [48] and Purcell et al. [49] in 1946, there have been several references in intact human cells and tissues for the study of living systems [50–52]. Soon after these attempts, Hoult and colleagues [53] showed that metabolism and pH could be measured in intact rat tissue in 1974. This research presaged the development of clinical MRS. In the 1980s, horizontal superconducting magnets were developed for in vivo spectroscopy, and in vivo measurements on live animals were successfully performed with MRS [54–57]. This application of relatively large bore high field magnets for in vivo spectroscopy is still a valuable innovation. A major challenge in performing measurements in a living organism is how to localize signals to an area of interest. Gordan and co-workers developed topical magnetic resonance in the 80 s of the twentieth century. The group utilized special field profiling coils to destroy magnetic field homogeneity other than in an area of interest only [55, 58]. In the same period, Acherman and co-workers introduced the surface coil, to localize signal to the immediate vicinity of a small RF coil and reduce the RF power [59]. This method considerably improved the signal-to-noise ratio. The potential of this new MR system to perform human measurements was quickly recognized, leading to systems that could accommodate a human limb for muscle investigations, and larger systems capable of measuring neonates and adult humans rapidly became available. The first whole-body high field magnets for MRS presaged the development of the 1.5 T magnets soon to be offered by major medical manufacturers. Due to the interest in muscle energetics, most of these early measurements were performed on ^31^P, but the potential to observe other nuclei, including ^1^H and ^13^C, with the potential to use ^13^C labeled compounds was quickly recognized. Changes in the resonant frequency of populations of nuclei give rise to the information content of MRS [60, 61].

**Fig. 3:**
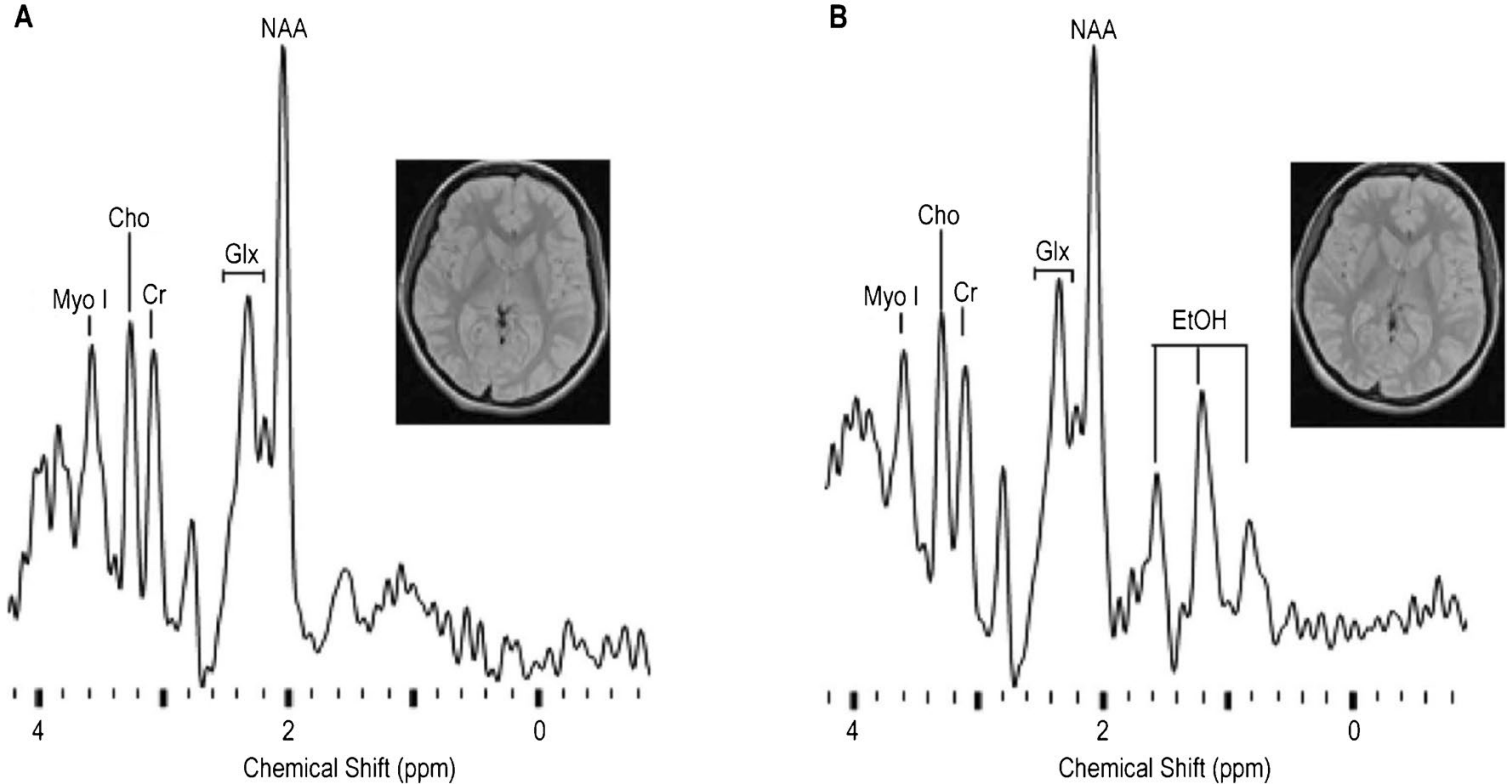
^1^H MRS spectrum and localizing MRI from a 0.56 cm^3^ voxel in the left putamen. Data acquired as part of a repeatability study. **A** Data collected in the first session. **B** Data collected from the second session when the volunteer was inebriated; triplet resonance of ethanol shown at 1.2 ppm [61]. Adapted with permission from ref 65, Copyright 2008 American Association of Physicists in Medicine

The unique part about MRS is its ability to provide real-time information about the chemical composition in a certain area of living tissues and organs in vivo. In complementary with MRI which presents imaging with detailed structural information, MRS measures fingerprint signals of specific molecules like metabolites and neurotransmitters. In a typical apparatus, MRS is performed with the same scanner and RF coil as MRI. With a specialized pulse sequence, MRS detects signals from specific molecules of interest. As shown in Fig. 2, a ^1^H MRS spectrum is collected accompanied by a localizing MRI scan from the brain of a volunteer in a repeatability study [61]. MRI scans show no obvious difference between the first (Fig. 3A) and the second (Fig. 3B) session; however, MRS results detect an appearance of ethanol in the second measurement. This lies along with the fact that the volunteer has had a late-night drinking. The combination of MRS with MRI allows researchers and clinicians to study the biochemical processes occurring in the body, enhance the diagnostic accuracy, and monitor the disease progression or treatment response, especially in brain metabolism, tumor characterization, and the study of medication effects.

### Metabolism study and clinical utilization of ex vivo and in vivo MRS

MRS yields real-time chemical information in living tissues and provides dynamic information for physiological responses. It can contribute to understanding metabolism in living samples and even humans. For instance, the liver is the central organ for lipid metabolism, fat storage, cholesterol synthesis, liponeogenesis, and production of triglycerides and lipoproteins. ^1^H MRS is the most effective method for the determination of liver fat non-invasively, and to monitor hepatic lipid content non-invasively on a large scale. With ^1^H MRS measurements, studies prove that interventions like low-calorie diet and dietary counseling combined with increased physical activity reduce hepatic steatosis and insulin resistance [62, 63]. Weight loss through gastric bypass also leads to dramatic changes in liver fat content [64, 65]. Hepatic fat analysis via ^1^H MRS is also quantifiable, and a previous study suggests that exercise increases the unsaturated fatty acids content in the liver [66]. However, the classification of saturated and unsaturated fatty acids is ambiguous since unsaturated resonance is in low abundance, and high-intensity water peak may interfere with the assignment.

#### MRS for dynamic measurements

**Fig. 4:**
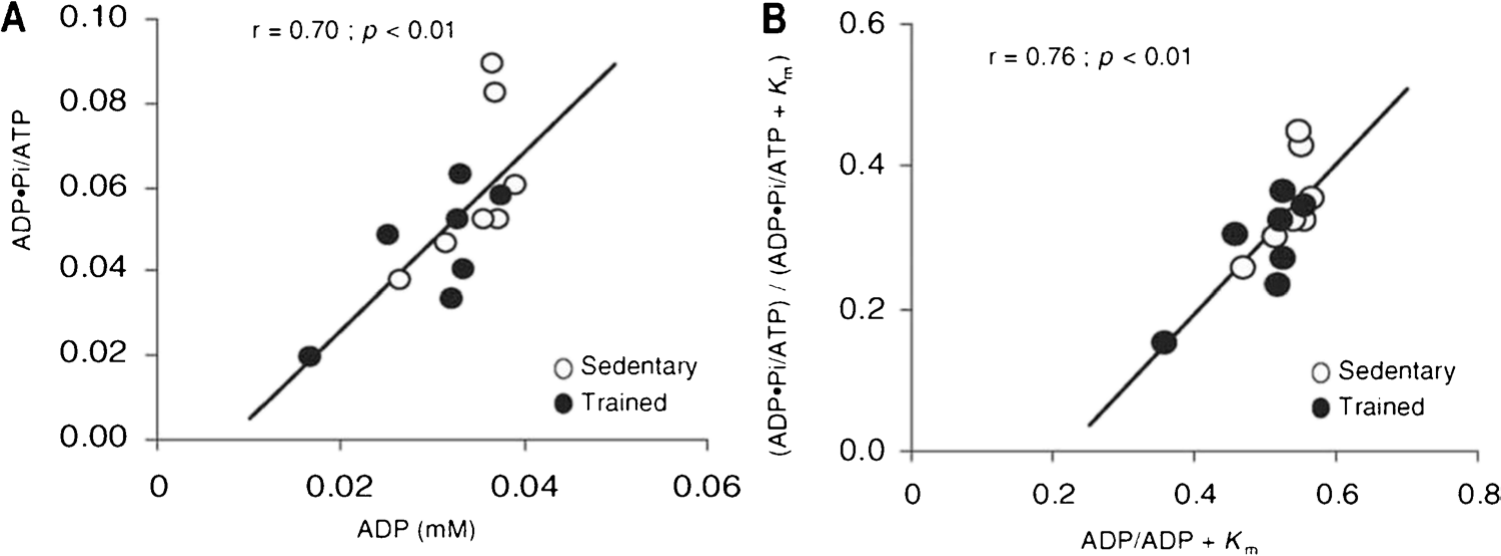
Linear relationship between different potential controllers of oxidative ATP synthesis in 14 subjects during a 6-min constant-load knee extension exercise. **A** Relationship between phosphate potential and ADP concentration averaged over the exercise period. **B** Relationship between affinity constant (Km = 0.11 mM) corrected phosphate potential and affinity constant (Km = 30 µM) perturbated ADP concentration [67]. Adapted with permission from ref 71, Copyright 2010 John Wiley and Sons

A great advantage of using MRS for metabolism study is to monitor dynamic information and response to a physiological stimulus. A classic study in this application is using ^31^P MRS tacking relative change of ATP in skeletal muscle during and after exercise. In 2011, Layec et al. [67] reported a study on muscle energetics during exercise and recovery. The study compared the rates of ATP production and energy cost in two groups of subjects with different training statuses, as shown in Fig. 4. MRS was used to quantify high-energy phosphate metabolites (such as PCr, Pi, and ATP) and pH in skeletal muscle non-invasively. Rates of ATPox and anaerobic ATP synthesis are therefore calculated through different metabolic pathways, including oxidative phosphorylation, anaerobic glycolysis, and ATP synthesis through the creatine kinase reaction. By comparing the results obtained from different methods, the study aimed to determine if the choice of method significantly affected the expected changes in energy production rates and mitochondrial functions. This information can provide valuable insights into muscle metabolism and help understand the impact of training on energy production pathways [67].

Acetylcarnitine can also be detected by MRS, especially with a long echo time of ^1^H MRS. Formed from the corresponding acetyl-CoA, acetylcarnitine plays a crucial role in transporting fatty acids into the mitochondria for energy production. In 2014, Lindeboom et al. [68] utilized long-echo time ^1^H MRS to measure skeletal muscle acetylcarnitine concentrations and investigated the relationship between acetylcarnitine concentrations, insulin sensitivity, and mitochondrial function in different groups of individuals, including endurance-trained athletes, lean sedentary subjects, obese sedentary subjects, and patients with type 2 diabetes mellitus (T2DM). Quantify with MRS, acetylcarnitine concentrations and PCr recovery rates in the skeletal muscle of the participants can be determined. Insulin sensitivity and PCr recovery rates between the different groups appear significant differences, with endurance-trained athletes showing the highest insulin sensitivity and mitochondrial function, while T2DM patients show the lowest. These results demonstrate that measuring acetylcarnitine concentrations with ^1^H-MRS is feasible on clinical MR scanners, and support the hypothesis that T2DM patients are characterized by a decreased formation of acetylcarnitine, possibly underlying decreased insulin sensitivity [68].

#### MRS in neurological study and diagnosis

In neurological research, MRS has also been applied to understand mechanisms of various neurological disorders, including measurements of brain metabolites, investigations of neurodegenerative diseases and brain tumors, and assessments of treatment responses. ^1^H MRS can quantitatively monitor a variety of metabolites in the brain, including N-acetyl-aspartate (NAA), total choline (tCho), creatine (Cr), myo-inositol (MI), lactate (Lac), and various amino acids. For example, NAA is synthesized from acetyl co-enzyme A and aspartate in neuronal mitochondria, and broken down by cytosolic deacetylation in oligodendrocytes. It diffuses along axons and is widely used as a marker for the degree of neuronal/axonal integrity of brain diseases [69]. Reduced NAA levels have been observed in cortical tissue from patients with Alzheimer’s disease (AD) and have demonstrated a correlation between NAA concentration and neuronal density [70]. In 2006, Godbolt et al. [71] found that metabolic changes were detectable in presymptomatic carriers of AD years before the expected onset of the disease. ^1^H MRS is utilized in the study to measure metabolic abnormalities in the brains of individuals with presenilin 1 (PS1) and amyloid precursor protein (APP) mutations, who have a nearly 100% risk of developing AD before they show any symptoms. The results revealed that the ratio of NAA, MI, and Cho to Cr was significantly reduced in presymptomatic mutation carriers relative to controls and that their magnitude was related to the proximity of expected age at onset [71]. Quantification of NAA and other metabolites can guide neurological diagnosis. However, the precise roles of metabolites like NAA are not well understood yet. ^1^H or other nuclei MRS can certainly help in investigations in this field.

#### MRS in cancer study and diagnosis

MRS is utilized in the diagnosis of various types of cancers since a major hallmark of cancer is its aberrant metabolism, especially ^1^H MRS. Tumor cells usually have abnormal metabolism, since they rely heavily on glycolysis, even in the presence of oxygen. This allows them to produce energy and building blocks for rapid cell division. In 2011, Wang et al. [72] investigated markers of pancreatic diseases and provided related data and experimental methods for the diagnosis of pancreatic diseases. ^1^H MRS was used to analyze the pancreatic juice from 15 patients, 10 of whom had pancreatic cancer and 5 had chronic pancreatitis with a mass in the head of the pancreas and tried to explore the markers of these diseases. One special experiment involved in this study was the 2D MRS. ^1^H-^1^H total correlation spectroscopy (TOCSY) was used to analyze the pancreatic juice sample and assign the resonance of various metabolites. A characteristic metabolite containing ethoxy group of alcoholic chronic pancreatitis was identified and expected to be used to distinguish pancreatic cancer from alcoholic chronic pancreatitis with a mass in the head of the pancreas [72].

Prostate cancer was also tested via ^1^H MRS. In 2014, Kobus et al. [73] investigated the presence of lactate (Lac) in patients with highly aggressive prostate cancer using in vivo ^1^H MRS with a semi-LASER sequence to optimize the detection. Seventeen patients were sampled and analyzed to determine the presence of Lac with respect to prostate cancer. Unfortunately, none of the identified tumors showed a convincing Lac signal. The low intensity of the Lac signal indicates its low concentration in highly aggressive prostate cancers. This was in contrast with the condition of brain tumors, which have been observed to have increased Lac signals ranging from 1.5 to 67.1 mM. However, the lack of a Lac signal does not necessarily mean that Lac is not produced by these tumors, but may suggest that Lac is either rapidly cleared from the prostate or produced in small amounts [73].

### Enhancing signal resolution and quality for MRS

Ex vivo NMR and in vivo MRS measurements are known for their ability to provide detailed information about the chemical composition of tissues and organs. However, the low resolution and low sensitivity of these two methods still hinder the detection of changes in target metabolites in tissues. Different approaches to improve signal resolution and quality have been explored and investigated.

#### High-resolution magic-angle-spinning nuclear magnetic resonance (HR-MAS NMR)

High-resolution magic angle spinning (HR-MAS) NMR spectroscopy is a powerful method for studying intact biological tissue. Standard ^1^H MRS or NMR analysis of living tissue is compromised for poor spectral resolution. The high-resolution magic angle spinning (HR-MAS) method can reduce MR spectral line widths and can be ideal for elucidating in vivo MRS observation [74, 75]. In HR-MAS NMR, the sample is spun at several kHz along a magic angle (54.7°) toward the direction of the static magnetic field. This efficiently reduces the residual dipolar interactions and the influence of magnetic susceptibility, leading to high-resolution NMR spectra [6]. Furthermore, it requires only small amounts of unprocessed samples and analyzes intact tissue samples directly to minimize information loss and artifacts introduced from the extraction [75].

**Fig. 5:**
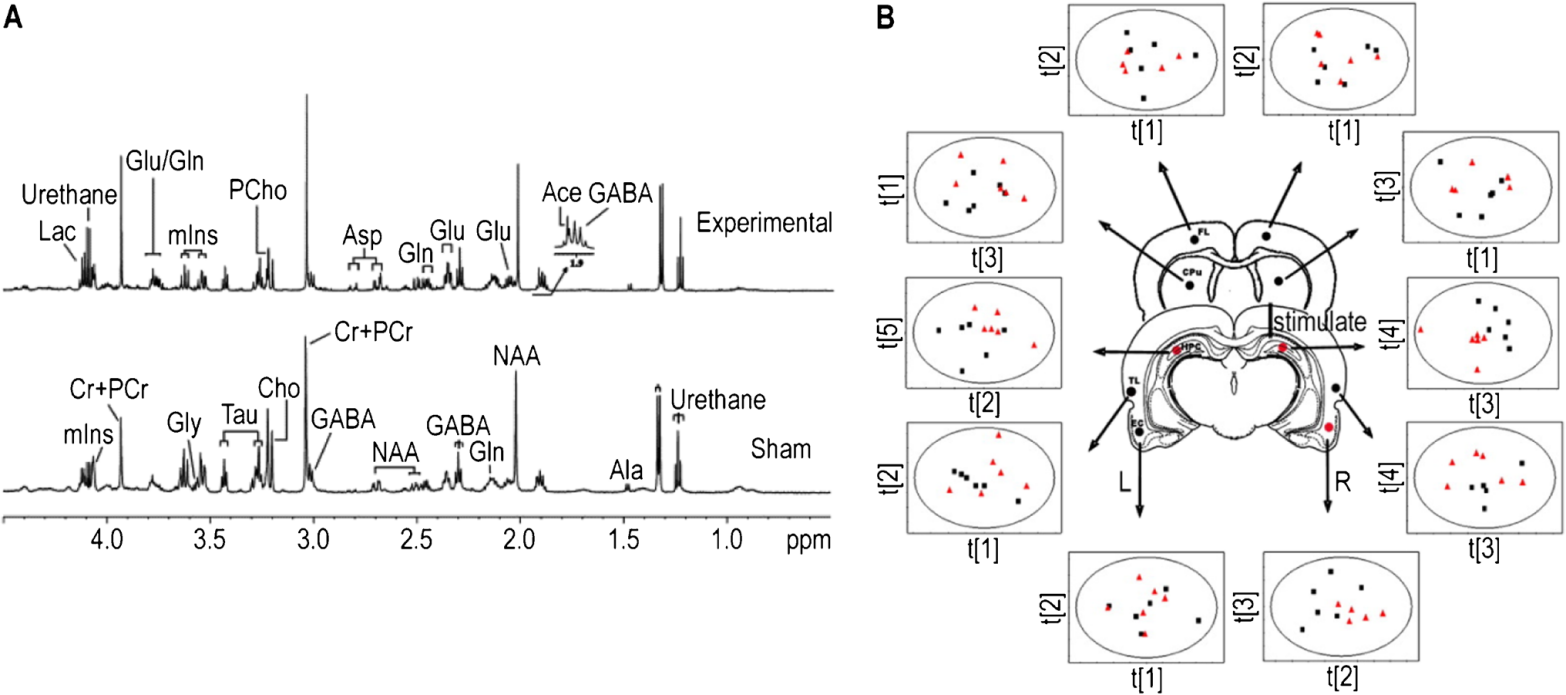
Identification and plot of major metabolites in the rat brain. **A** Label of major metabolites from HR-MAS ^1^H NMR spectra. **B** Score plots based on HR-MAS ^1^H NMR spectra of intact tissues from areas in two hemispheres [8]. Adapted with permission from ref 8, Copyright 2008 Elsevier

With this approach, we investigated the metabolic changes in temporal lobe structures in an early stage of epilepsy. Metabolites in bilateral hippocampi, entorhinal cortices (ECs), and temporal lobes (TLs) of rats were measured and analyzed, as shown in Fig. 5A. Results showed significant differences in metabolic profiles between the experimental and sham groups in the bilateral hippocampi and the ipsilateral EC. Significant increases in total creatine were observed in the ipsilateral hippocampus and alanine in the ipsilateral TL in the experimental group. Some metabolite levels were also disturbed in the bilateral HPC-EC loops. Principal component analysis (PCA), a multivariate data analysis method, was applied to the HR-MAS NMR data. As shown in Fig. 5B, metabolic profile variations in these brain areas might be different between the experimental and sham groups. Using HR-MAS NMR, the study addressed metabolic information about temporal lobe structures and contributed to a better understanding of epileptic abnormalities at an early stage [8].

**Fig. 6:**
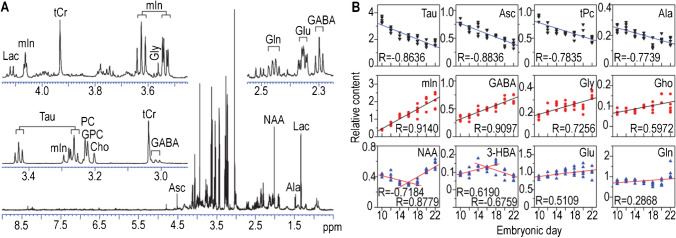
Identification and tracking of major metabolites in the cerebral tissue of a chick embryo during incubation. **A** Assignment of major metabolites in HR-MAS ^1^H NMR spectra. **B** Changes in the relative concentration of major metabolites in cerebral tissue with the development of chick embryo [6]. Adapted with permission from ref 6, Copyright 2015 Public Library of Science

Later on, HR-MAS NMR was also applied to analyze changes in cerebral metabolites during chick embryo incubation. As shown in Fig. 6A, multiple important biological molecules like Lac, tCr, Gaba, glu, and gln were identified using HR-MAS ^1^H NMR with high efficiency in broad peak suppression, excellent baseline, and resolution, which are comparable to a conventional solution NMR spectrum. This demonstrated that HR-MAS NMR is a powerful approach for studying metabolites in intact tissue without any sample pretreatment. Multiple major metabolites signaling various pathways exhibited significant changes in relative concentration during the incubation period, as shown in Fig.  6B. These metabolites observed can be classified into three categories: neurotransmitters, nutrition sources, and neuronal or glial markers, and the changes in metabolite concentrations were interconnected both within and between these three categories. Furthermore, the number and size of brain neurons were also found to be associated with the concentration changes during incubation, which suggests an interconnection between changes in cerebral metabolites and the development of the embryonic brain. This study provides valuable biochemical and neurochemical information about the development of the embryonic brain and highlights the interconnected nature of cerebral metabolite changes during this process [6].

#### Hyperpolarization methods

Hyperpolarization methods are utilized to enhance the signal-to-noise ratio and improve the sensitivity of measurements allowing for the detection of low-concentration metabolites, such as lactate, in real time and providing valuable information about the dynamics of cell energy metabolism. Hyperpolarization involves increasing the polarization of the nuclear spins in the sample and can be achieved by using techniques such as dissolution dynamic nuclear polarization (dDNP) [76], parahydrogen-induced hyperpolarization (PHIP) [77], and more. dDNP targets samples in solution [78–81]. Rely on the microwave irradiation in a magnetic field [2, 79]. Electron spin polarization, which is significantly higher than that of nuclei, can be transferred to the surrounding nuclear spins, resulting in a significant increase in the signal intensity of the nuclei of interest [82–84]. Sensitivity enhanced by dDNP allows for a more detailed analysis of solubilized small molecules and biological systems [85] and has provided significant assistance in metabolism studies with heteronuclear NMR spectroscopy [86]. PHIP on the other hand, uses symmetry-breaking catalysts to activate and hyperpolarize singlet spin isomer or paraisomer of H_2_, hence enhancing magnetic resonance signals dramatically [87]. The addition of p-H_2_ with catalysis such as organometallic complexes to unsaturated substrates can make them hyperpolarized [88].

In 2010, Wijnen et al. [89] studied glucose metabolism in human brain tumors using ^13^C MRS. The optimized ^13^C MRS method along with an intravenous infusion of [1-^13^C]glucose under euglycemic conditions was utilized to measure the dynamic conversion of glucose into its metabolic products in glioma tissue. The study included measurements in two different brain regions in two patients with brain tumors. The results revealed increased glucose uptake and lactate production in the tumor tissue compared to the healthy tissue. dDNP method enhanced signal intensity, and improved the ^13^C MRS approach to assess metabolic activity in human tumor tissue [89]. For MRS targeting nuclei in low natural abundance like ^13^C, signal enhancement significantly widens its utilization.

**Fig. 7:**
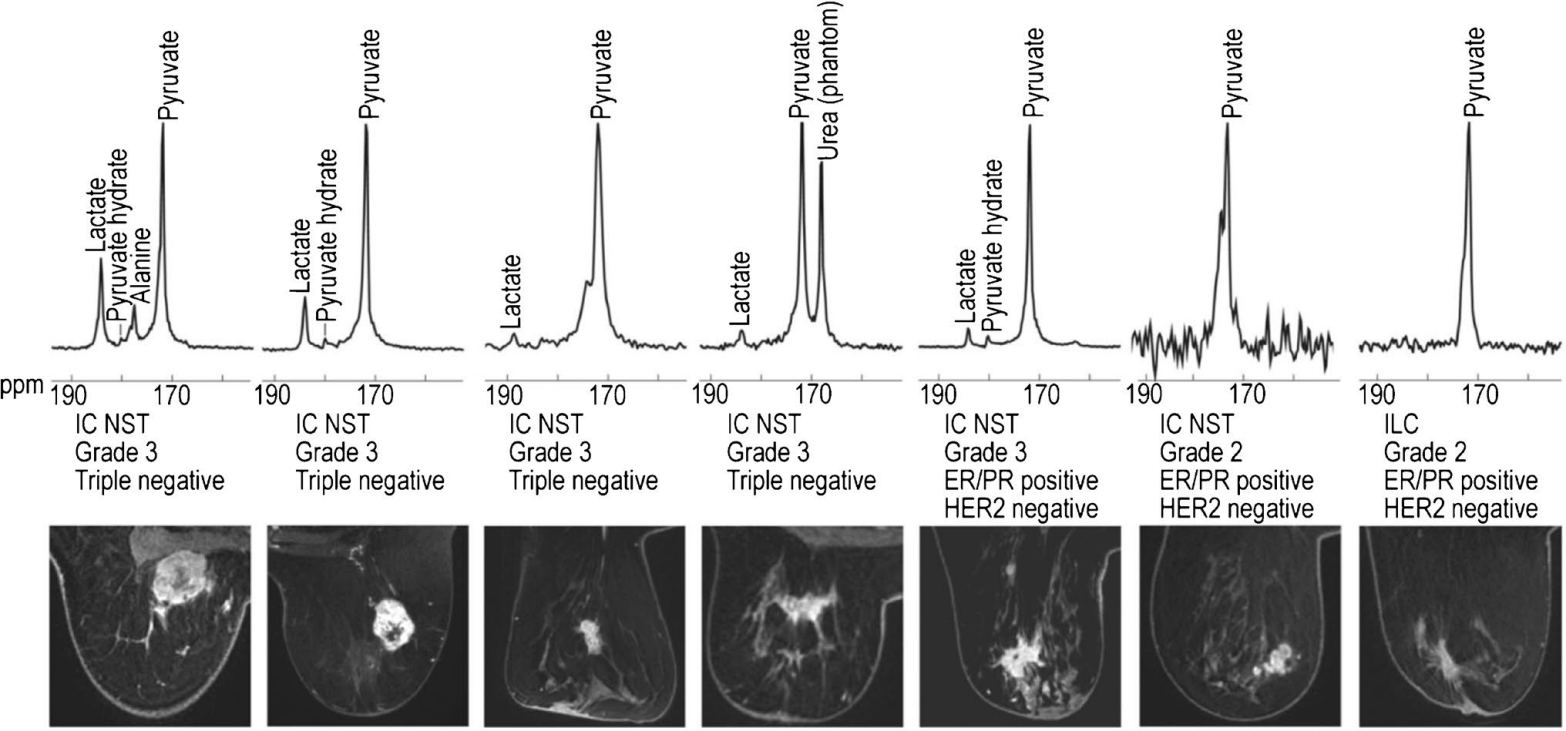
Hyperpolarized ^13^C MRS spectra (top) and images (bottom) of slices of tumors from the patients [90]. Adapted with permission from ref 94, Copyright 2020 United States National Academy of Sciences

In 2020, Gallagher et al. [90] assessed the exchange of hyperpolarized ^13^C label between injected [1-^13^C]pyruvate and the endogenous tumor lactate pool to track tumor metabolism in breast cancer via magnetic resonance spectroscopic imaging. A solution of hyperpolarized pyruvate was injected into patients with breast cancer at different stages. The resulting metabolic products are then monitored using magnetic resonance spectroscopy imaging (MRSI). As shown in Fig. 7, the intensity of ^13^C-pyruvate and ^13^C-lactate signals were both enhanced, providing discernable signals in different tumor samples. Corresponding lactate signal is mainly derived from the intracellular compartment at early time points, while extracellular space becomes the substantial location where lactate conjugates at later time points. Hyperpolarization-enhanced ^13^C MRSI enables tracking of the tumor metabolism, providing valuable information to further stratify breast cancer patients and guide treatment options [90].

**Fig. 8:**
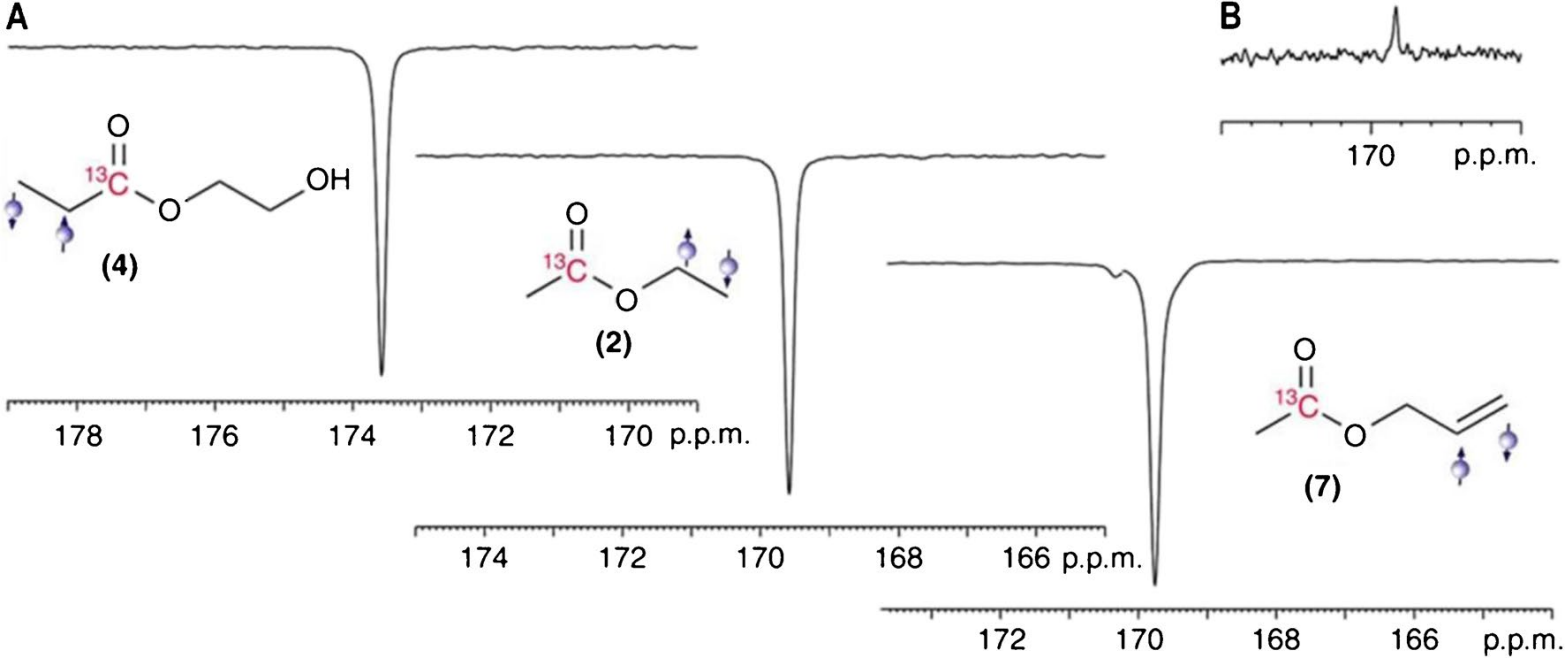
^13^C carboxylate hyperpolarization on different types of molecules. **A** Similar hyperpolarization obtained despite different positions p-H_2_ was introduced. **B** The thermal equilibrium spectrum of acetic acid allyl ester was used as reference [91]. Adapted with permission from ref 92, Copyright 2015 Nature Portfolio.

In 2015, Reineri et al. [91] reported enhancement of the ^13^C NMR signal of acetate and pyruvate, both of which are widely studied metabolites, via the PHIP method. Precursors containing a hydrogenable functionality were used and the parahydrogenation reaction was carried out in acetone-d_6_. Polarizations were achieved for the carboxylate signals of the allyl ester in pyruvate by applying magnetic field cycling. Hydrolysis and phase separation were then quickly carried out to obtain the hyperpolarized metabolite. As shown in Fig. 8A–C, the addition of p-H_2_ significantly enhanced the ^13^C signal of different types of molecules. This successful hyperpolarization of acetate and pyruvate demonstrated offers a cheaper and easier method for hyperpolarization [91].

#### Isotopic enrichments

For nuclei such as carbon and nitrogen, which are naturally present in low abundance [92], the low signal intensity in MRS may obscure valuable information and impede the study of metabolic pathways. However, MRS targeting these nuclei possesses unique attributes in terms of distinctive chemical specificities, which may assist in the detection and quantification of metabolites. The enrichment of isotopes facilitates accurate measurements of metabolites of interest, resolving the fluxes of metabolic pathways. The process of isotopic enrichment commences with the administration of isotopically labeled substrates, wherein the labeled molecules are infused into the experimental systems. Subsequently, metabolites can be monitored via MRS techniques and extracted at various time points. The analysis of isotopically labeled signals then culminates in the generation of mathematical models that estimates the rates of metabolic flux in the studied pathways [93–95]. Isotopic enrichment provides insights into kinetic studies of metabolic flux, thereby assisting in the understanding of metabolic behavior and pathways.

**Fig. 9:**
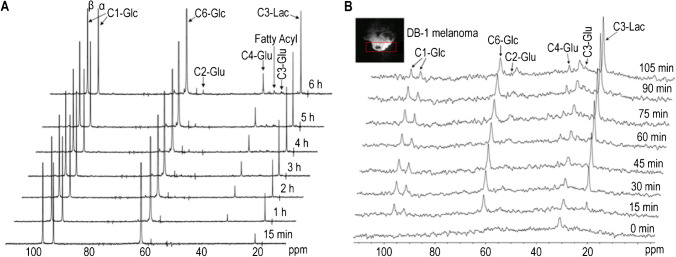
Time course ^13^C MRS exhibiting glutamate (glu), lactate (lac), glucose (glc), and long-chain fatty acid (fa), over 6 h, where [1,6-^13^C_2_] glucose was used as the substrate for ^13^C-labeling. **A** Time course ^13^C MRS of perfused DB-1 melanoma cells in bioreactor. **B** Time course ^13^C MRS of a DB-1 melanoma xenograft [96]. Adapted with permission from ref 100, Copyright 2016 Frontiers

In 2016, Shestov et al. [96] investigated several facets of cancer cell metabolism, encompassing the fluxes of glycolysis, the pentose phosphate pathway, and glutaminolysis in DB-1 melanoma cells. Isotopically enriched ^13^C MRS was employed to monitor and quantify metabolite patterns, subsequently constructing a metabolic network model to estimate the fluxes through key pathways. The cells under investigation were administered ^13^C-labeled substrates, and the distribution of isotopic labels among diverse metabolites was tracked via ^13^C MRS. As shown in Fig. 9, temporal alterations in the isotopic labeling system, such as the elevation of glutamate and lactate, were analyzed. Time course trajectories of concentration changes in different metabolites were graphed to estimate the fluxes through various metabolic pathways. Utilization of isotopic enrichment allows accurate measurements of metabolites at low concentrations and assists in the differentiation of metabolites involved in corresponding processes [96].

In 2012, Cudalbu et al. [97] studied the role of brain glutamine synthetase in the detoxification pathway for hyperammonemia. ^15^N-labeled ammonium chloride was infused with rat brains to perform in vivo ^15^N and ^1^H MRS, and to measure various metabolic fluxes associated with cerebral glutamine metabolism. Subsequently, a mathematical model was constructed based on the time courses of both regular and isotopically labeled glutamine, enabling the determination of its net synthetic rate. The observed increase in glutamine synthetase and net glutamine accumulation under hyperammonemic conditions corroborated the involvement of the detoxification metabolic pathway of cerebral ammonia. The isotopic enrichment of ^15^N enhanced detection sensitivity, thereby enabling direct measurements of ^15^N incorporated glutamine and facilitating the accurate quantification of metabolite concentrations [97].

## Challenges and perspectives of NMR and MRS spectroscopy in metabolism studies of living systems

NMR and MRS spectroscopy have shown great applications in metabolism studies. Like the examples discussed in the previous chapters, these spectroscopy methods have assisted in the analysis and tracking of metabolites, identification of metabolic changes, and diagnosis and progress monitoring of diseases clinically. However, improving the sensitivity and resolution of NMR spectroscopy is still a major challenge nowadays. Special and complicated methods have to be applied to generate qualified signals originating from metabolites of interest. This increases the effort and cost of metabolism studies.

### Challenges and perspective of in-cell NMR

NMR has a number of advantages such as non-destructive, with high reproducibility, and highly quantitative, which provides a powerful tool for the study of metabolism inside living cells. However, it is much more challenging for metabolism studies in living cells than in vitro studies. One challenge with in-cell NMR samples arises from the need to keep the cells healthy and metabolically stable during NMR data acquisition. It is necessary for in-cell NMR studies when long acquisitions are required, especially for environmentally sensitive cells. Besides, the broadening effects of field inhomogeneity in bio-samples, which results in poor peak line width and resolution, significantly reduce the ability of NMR to identify and quantify the metabolites accurately. Therefore, further development in methods for keeping cell health status in the NMR tube as well as NMR techniques to improve detection limits and resolution should further empower NMR in cellular metabolism studies.

### Challenges and perspective of MRS

Currently, one big limitation of MRS, both in vivo and ex vivo, is the low signal intensity, even in a high magnetic field. Because of the low concentration of biochemicals in tissue, the spatial resolution of MRS cannot satisfy detailed anatomical analysis. Moreover, it is difficult to increase isotopic concentrations of targeted nuclei, especially for those in low natural abundance. In more and more up-to-date studies, the hyperpolarization method has been introduced to MRS for signal enhancement, and examples are discussed in previous chapters. Hyperpolarization significantly increases the signal intensities of analytes. The sensitivity gain of NMR signals permits the detection of analytes or nuclei which suffer from poor sensitivity due to their low natural abundance, low gyromagnetic ratios, or large anisotropic interactions. However, complicated sample preparation requires extra time and effort. Such methods for signal enhancement are also limited by factors such as solubility, electron-electron distance, and electron spin relaxation time of the polarizing agents used. For example, the lifetime of hyperpolarization decays towards thermal equilibrium with the nuclear spin lattice relaxation time, which can be as short as the time scale of miliseconds^81,(90)^. Furthermore, hyperpolarized agents usually require injection to function. This goes in conflict with the utilization of MRS as a non-invasive tool and also introduces higher costs and risks than non-invasive analytical methods. Development of techniques like endorectal coils, dual-frequency pulses, and outer volume saturation slabs to improve lipid suppression and localization of MRS, but these are time consuming and more importantly subjective. An easily handled method improving the signal intensity of MRS is in demand.

MRS is capable of providing spatial-spectral information. Examples discussed in the previous chapters address the significant role MRI plays in the identification of metabolites in real time and in living tissues. This serves important clinical applications in metabolism studies, neurological studies, and disease diagnoses like neurodegenerative diseases and cancers. With the enhancement of MRS signal quality, the utilization of this spectroscopy will greatly level up the current stage of metabolism studies.

## Conclusion

Metabolism study illustrates chemical reactions that occur in living organisms to sustain life. Understanding metabolism is key to understanding life [98]. Over the past decades, multiple NMR techniques have been used to assist the studies, including in vitro in-cell NMR and in vivo MRS. In-cell NMR offers information on metabolites and proteins in the physiological intracellular environment at the atomic level. In vivo MRS provides qualitative measurements of metabolites under study in specified areas of living organisms. Measurements in real-time and native environments reveal active information in metabolism studies. Continued developments in the future are expected to further enhance the signal quality and application range of these NMR techniques.
